# Transperitoneal Calcium Balance in Anuric Continuous Ambulatory Peritoneal Dialysis and Automated Peritoneal Dialysis Patients

**DOI:** 10.1155/2013/863791

**Published:** 2013-07-09

**Authors:** Chieko Hamada, Yasuhiko Tomino

**Affiliations:** Division of Nephrology, Department of Internal Medicine, Juntendo University Faculty of Medicine, 2-1-1 Hongo Bunkyo-ku, Tokyo 113-8421, Japan

## Abstract

*Backgrounds*. Calcium (Ca) and bone metabolism in continuous ambulatory peritoneal dialysis (CAPD) and hemodialysis (HD) patients show a remarkable difference depending on dialysis modalities. The levels of serum Ca and phosphate (P) in HD patients fluctuate contributing to the intermittent and rapid removal of plasma solute unlike in CAPD. Characteristics of plasma solute transport in automated peritoneal dialysis (APD) patients are resembled with that in HD. The purpose of the present study was to examine the difference of transperitoneal Ca removal between APD and CAPD anuric patients. *Subjects and Methods*. Twenty-three APD anuric patients were enrolled in this study. Biochemical parameters responsible for transperitoneal Ca removal in 24-hour and 4-hour peritoneal effluents were analyzed on CAPD and APD. *Results*. Transperitoneal Ca removal on APD was smaller compared with that on CAPD. The Ca removal was related to the ultrafiltration during short-time dwell. Decrease of the Ca removal during NPD induced by short-time dialysate dwell caused negative or small Ca removal in APD patients. The levels of intact PTH were increased at the end of PET. *Conclusion*. It appears that short-time dwell and frequent dialysate exchanging might suppress the transperitoneal Ca removal in anuric APD patients.

## 1. Introduction

 Bone disease is one of the serious complications in chronic dialysis patients. Adynamic bone disease and secondary hyperparathyroidism are associated with not only viability and quality of life (QOL) but also mortality in long-term dialysis patients. It is generally considered that Ca and bone metabolism between peritoneal dialysis (PD) and hemodialysis (HD) patients provided remarkable differences according to dialysis modalities. Hemodialysis patients undergo rapid and intermittent removal of phosphate, uremic toxins and excess body fluid from sera, and influx or efflux of Ca influent in such metabolism [1–3]. Higher serum Ca levels and continuous glucose loading occur, which may lead to a higher incidence of adynamic bone in CAPD patients compared with that in HD patients [4, 5]. Patients with very low parathyroid hormone (PTH) level had a higher mortality rate after adjustment for age, gender, diabetes, and dialysis vintage [6]. The turnover of bone remodeling in PD patients is lower than that in HD patients [4, 5]. Using 3.5 mEq/L Ca dialysate in HD, Ca removal demonstrated a negative balance [7, 8]. It is recognized that Ca mass transport in CAPD patients depends on the following factors: Ca concentration in the dialysis fluid, starting plasma Ca and P concentrations, and ultrafiltration, which removes the nonprotein bound diffusible calcium [9–12]. Characteristics of plasma solute transport in patients with automated peritoneal dialysis (APD) are resembled with that of HD. Effects of APD on residual urinary volume compared with that in CAPD were reported [13, 14]. It is postulated that a short-time dwell of peritoneal dialysate may lead to differences in the transperitoneal Ca balance and PTH secretion between APD and CAPD patients.

 The objective of the present study was to evaluate differences of transperitoneal Ca balance between APD and CAPD patients without residual renal function.

## 2. Subjects and Methods

### 2.1. Patients

Twenty-three patients (15 males and 8 females) took a standard peritoneal equilibrium test (PET), and 24-hour peritoneal effluents (PE) were collected on CAPD for the determination of solute kinetics every year. Transperitoneal balance studies of calcium (Ca) were examined for these anuric patients treated by PD. All PD patients provided informed consent to participate in this study. Most of the measurements are performed in the routine of our hospital laboratory. The transperitoneal Ca balance was calculated according to the formula for peritoneal mass transfer as follows:

(1) Concentration  of  Ca×Drained  volume   −Concentration  of  Ca×Infused  volume.

A routine peritoneal equilibration test (PET) using 2.5% dextrose PD solution was performed after the initiation of PD in new patients and every 12 months thereafter. PET results were used for the patients in order to choose the optimized PD prescriptions. PD effluents were obtained just before the PET and at 4 hours as the long-time and short-time dwell samples. In uremic patients and non-CKD patients, family members gave their consent in writing. The Ethics Committee of Juntendo University Faculty of Medicine approved this study including evaluation of peritoneal morphology and peritoneal function.

Statistical analysis consisted of both simple and multiple linear regressions. Comparisons between the two groups were made using Mann-Whitney test. Relationships between ultrafiltration and transperitoneal Ca removal were analyzed with Spearman's correlation coefficient test. A value of *P* < 0.05 was regarded as significant. Data were expressed as mean ± standard deviation (SD).

## 3. Results

### 3.1. Characteristics of CAPD and APD Patients

Basic characteristics of participants are shown in Table 1. Mean age of patients was 51.1 ± 14.9 years old. Mean duration of PD was 78.7 ± 35.9 months. Noncalcium-based phosphate binders were not administered in all participants. The D/P ratio of creatinine in APD patients was 0.68 ± 0.13. Dairy average dosage of CaCO_3_ was 3.44 ± 2.57 g. Average dialysate Ca concentration in APD patients was 6.10 ± 0.94. Eleven APD patients used low-calcium PD solution in order to suppress elevation of serum Ca. Icodextrin PD solution was not used.

### 3.2. Calcium Balance in 24-Hour Collected Peritoneal Effluents

**Figure 1:**
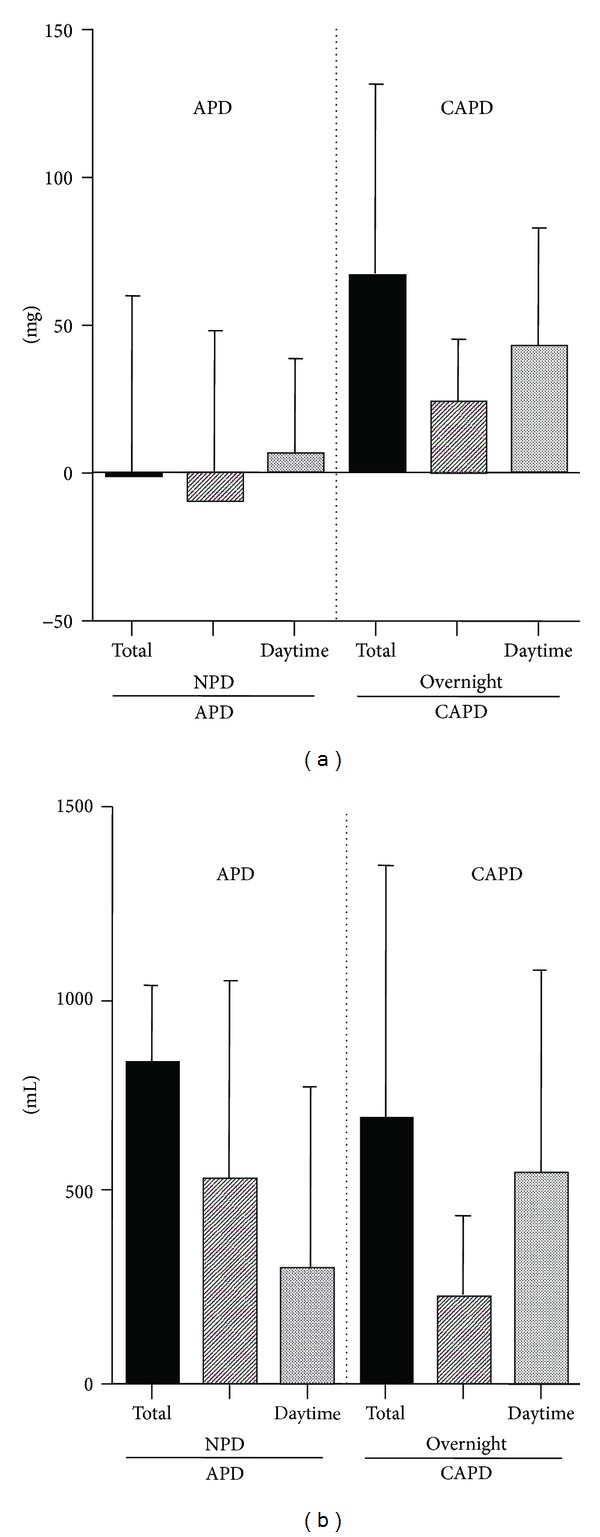
(a) Transperitoneal Ca balance and (b) ultrafiltration on APD and CAPD in same PD patients. Black bars show total Ca balance in 24 hours. Diagonal bars show transperitoneal Ca removal in NPD or overnight dwell. Dots bars show transperitoneal Ca removal in daytime dwells.

Eleven peritoneal effluents (PE) for 24 hours on APD and 23 PEs on CAPD were collected in the same patients (Table 2). There was no significant difference in the Ca concentration of PD solution between APD and CAPD. Ca removal on APD was significantly smaller than that in CAPD. Small molecule substance removal quantities were independent on the PD option and dwell volume. We obtained 24-hour collected PE on APD and CAPD on consecutive days in 23 PD patients. 1.37 ± 63.18 mg of Ca was absorbed from PD solution on APD, whereas 67.1 ± 58.6 mg of Ca was removed on CAPD (Table 3). Calcium was removed on CAPD; however, the Ca removal indicated zero on APD, using the same Ca concentration PD solution and removing the same amount of body fluid, respectively, (*P* < 0.01, Figure 1(a)). During night peritoneal dialysis (NPD), Ca was absorbed from PD solution (Figures 1(a) and 1(b)).

### 3.3. Relationship between Ultrafiltration and Intact PTH on PET

Calcium removal in PD patients using 2.5% dextrose dialysate containing 7.0 mg/dL of Ca is shown in Table 4. The ultrafiltration was 311.9 ± 137.7 mL. Calcium concentration in peritoneal effluent was markedly decreased at 4 hours. There was no significant change in the levels of serum Ca. The levels of intact PTH were remarkably increased at 4 hours of PET (from 170.2 ± 161.5 pg/mL to 201.1 ± 181.3 pg/mL, *P* < 0.002).

### 3.4. Relationship between Transperitoneal Ca Removal in PE and Ultrafiltration in Short-Time Dwell and Long-Time Dwell

**Figure 2:**
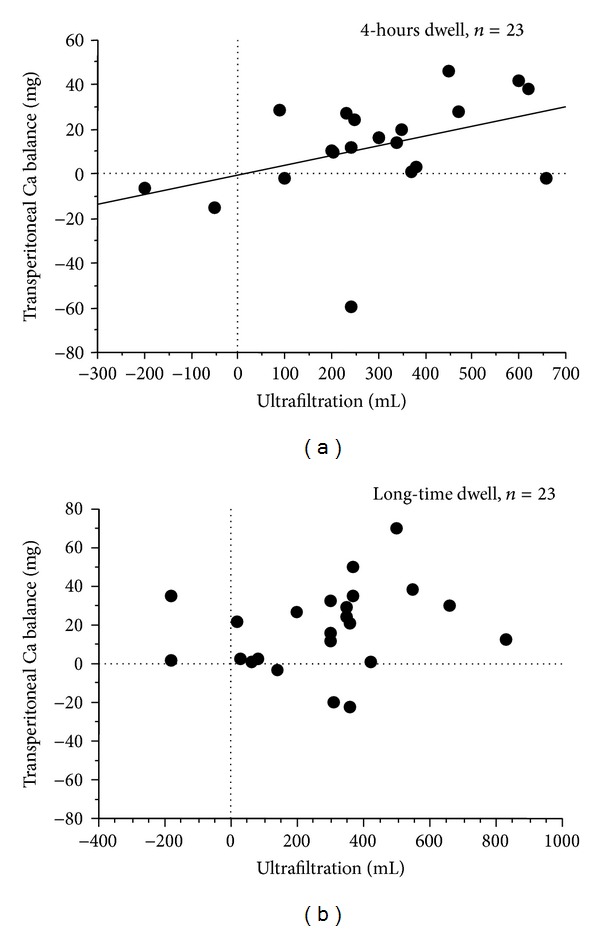
Relationship between transperitoneal Ca removal and ultrafiltration in short-time dwell and long-time dwell. (a) In 4-hour dwell. (b) In long-time dwell.

Transperitoneal Ca removal and ultrafiltration showed a significant positive relationship both in short-time and long-time dwell (Figure 2, coefficient value = 0.367, *P* < 0.02). Calcium removal was related to ultrafiltration in short-time dwell. Meanwhile, there was no relationship between Ca removal and ultrafiltration in long-time dwell. 

## 4. Discussion

We demonstrated that transperitoneal Ca removal in APD patients without residual renal function was smaller, compared with that in CAPD patients. The transperitoneal Ca removal was dependent on the PD modality. In the same patients, the Ca removal in APD was significantly lower than that in APD. The solute and body fluid kinetics have been shown to be quite different between CAPD and HD [2, 3]. Factors influencing calcium removal during dialysis are as follows: (1) ultrafiltration rate, (2) concentration of serum ionized calcium at starting of dialysis session, (3) concentration of serum inorganic phosphate at the start, and (4) concentration of dialysate calcium [9–12, 15, 16]. The levels of serum Ca and phosphorous in HD patients fluctuated, contributing to intermittent and rapid removal of solute and body fluid; meanwhile, constant solute and body fluid kinetics were major characteristics of CAPD therapy. Exchanges and removal of minerals and small molecule solutes were rather drastic during NPD and steady during day-time dwell in continuous cyclic PD (CCPD) patients. Since dialysate Ca concentration influences Ca balance during HD and CAPD, active vitamin D and calcium-containing phosphate binders regulating serum Ca concentration require a reduction in dose, from 1.75 to the more physiological dialysate Ca concentration [1, 7, 8, 17–19]. 

The present study showed that the dwell time and ultrafiltration played crucial roles in transperitoneal Ca removal in PD patients. Ca removal in long-time dwell was independent on ultrafiltration, whereas ultrafiltration increased Ca removal in short-time dwell. According to the results, it appears that smaller amount of Ca-containing phosphate binder in APD patients can be administrated compared with that in CAPD patients. The average concentration of dialysate Ca was 6.1 mg/dL, and all participants used low-calcium dialysate at least one or two bags to prevent the elevation of serum Ca. The level of PTH markedly increased during PET, but not the elevation of PTH (data not shown) in this study. However, the slight Ca change contributing to ultrafiltration might be a stimuli of PTH secretion.

## 5. Conclusions

This study showed the characteristics of the Ca removal in anuric APD patients as being somewhere between that of CAPD and HD. Short-time dwell and frequent dialysate exchanges during NPD suppressed the transperitoneal Ca removal in APD patients; it appears that the levels of serum Ca increase easily in anuric APD patients using calcium-containing phosphate binder.

## Figures and Tables

**Table 1:** Characteristics of participants.

Number of patients	23
Age (years)	51.5 ± 14.9
PD duration (months)	78.7 ± 35.9
Serum creatinine (mg/dL)	11.7 ± 2.48
Serum calcium (mg/dL)	10.0 ± 0.66
Serum phosphate (mg/dL)	5.07 ± 1.11
Intact PTH (pg/mL)	178.3 ± 180.4
Weekly Ccr (L/week)	51.9 ± 7.48
D4/P cre	0.68 ± 0.13
CaCO_3_ (g/day)	3.44 ± 2.57
Vitamin D3 (*μ*g/day)	0.03 ± 0.07
Dialysate Ca concentration (mg/L)	6.10 ± 0.94

Mean ± SD, Cre: creatinine, PET: peritoneal equilibration test, PTH: parathyroid hormone, and Ccre: creatinine clearance.

**Table 2:** Twenty-four-hour collected peritoneal effluent on APD and CAPD in same PD patients.

	Dwell volume (mL)	Drain volume (mL)	Ca of PDS (mg/dL)	24-hour collected PE				
				UN (mg)	Cre (mg)	TP (mg)	Ca (mg)	iP (mg)
APD *n* = 11 samples	14222.2 ± 1371.8	15101.0 ± 1356.5	5.9 ± 0.7	4911.7 ± 1280.8	869.4 ± 264.7	4.5 ± 2.1	9.9 ± 38.7	378.2 ± 75.0
CAPD *n* = 23 samples	8200.0 ± 462.6*	9190.0 ± 698.0*	6.0 ± 1.8	4783.4 ± 1263.2	927.7 ± 268.0	7.0 ± 3.2*	111.3 ± 107.2*	355.0 ± 126.8

Mean ± SD, *P < 0.001, **P < 0.02 versus APD.

PDS: peritoneal dialysis solution, UN: urea nitrogen, Cre: creatinine, TP: total protein, and iP: inorganic phosphate.

**Table 3:** Transperitoneal Ca balance and ultrafiltration on successive PD treatment in the same patients.

	Ca concentration of dialysate (mg/L)	Ultrafiltration (mL)	Transperitoneal Ca balance (mg)
APD	5.84 ± 0.72	832.44 ± 225.66	−1.37 ± 63.18
CAPD	6.00 ± 0.90	773.75 ± 595.03	67.12 ± 58.63*

Mean ± SD, *P < 0.05 versus APD, *n* = 23 patients.

**Table 4:** Effluent and serum Ca and parathyroid hormone on PET.

		At the start	At 4 hours
PE volume	(mL)	2116.7 ± 65.8	2428.6 ± 137.7*
Ca concentration of PE	(mg/dL)	3.90 ± 0.14	3.36 ± 0.21^#^
Ca concentration in sera	(mg/dL)	9.44 ± 0.70	9.41 ± 0.60
Intact PTH	(pg/mL)	170.2 ± 161.5	201.1 ± 181.3**

Mean ± SD, *P < 0.01 versus at the start, **P < 0.002 versus at the start, ^#^ *P* < 0.0001 versus at the start, *n* = 23.

PET: peritoneal equilibrium, PE: peritoneal effluent, Ca: calcium, and PTH: parathyroid hormone.
